# Does the trade of medical products contribute to promoting human development?—An empirical analysis based on data from China and RCEP countries

**DOI:** 10.3389/fpubh.2025.1650225

**Published:** 2025-08-13

**Authors:** Liang Wu, Wenxia Liu, Jianhua Zhou, Dan Zhou, Hanjie Xiao

**Affiliations:** School of Economics and Management, Huzhou University, Huzhou, China

**Keywords:** RCEP, HDI, medical products, trade efficiency, stochastic frontier gravity model

## Abstract

**Background:**

As the RCEP (Regional Comprehensive Economic Partnership) Agreement deepens, the role of medical product trade in safeguarding regional public health security and promoting economic development has become increasingly prominent.

**Methods:**

Based on data from 2004 to 2023, this study employs the stochastic frontier gravity model and benchmark regression model to explore the impact of China’s medical product export efficiency on the development indices of RCEP countries.

**Results:**

The findings are as follows: (1) China’s medical product exports to RCEP countries exhibit simultaneous scale expansion, structural differentiation, and market concentration, with ASEAN, Japan, and South Korea as core markets and mid-to-low-end products dominating the export structure; (2) Trade efficiency evolution reflects dual mechanisms of institutional environment and technological capabilities, where governance levels and health system soundness optimize efficiency, while excessive liberalization and tax burdens increase friction costs; (3) Enhanced export efficiency indirectly promotes human development in RCEP countries by strengthening basic health service coverage, forming a “trade-health-development” transmission mechanism.

**Conclusion:**

To enhance export efficiency and achieve synergistic development, we propose: (1) For policymakers: Deepen institutional coordination and establish a “trade-health” collaborative governance framework to amplify public health dividends; (2) For exporters: Implement a tiered market strategy and strengthen technological innovation to enhance industrial resilience.

## 1 Introduction

In the context of profound changes in the global health governance system, the trade efficiency of medical products, as a key strategic resource for maintaining public health security and driving economic structural transformation (1), is not only of decisive significance for the resilience of regional supply chains, but also has a far-reaching impact on the global human development pattern. Accompanied by the sustained and rapid growth in the demand for medical products (2, 3), how to promote the synergistic allocation of inter-regional medical resources and the balanced development of human well-being through the optimization of trade efficiency has become an urgent and realistic issue to be resolved.

Although existing studies on trade have covered multiple dimensions such as trade network analysis (4), quantitative assessment of non-tariff barriers (5) and exploration of influencing factors (6), forming a relatively mature research paradigm, there are still significant shortcomings in the specific area of medical products. First, previous studies have mostly focused on the trade of bulk commodities, and have paid significantly less attention to medical products, a category with special attributes and an important strategic position. Second, the role of health factors as a key mediating variable has not been fully considered when analyzing the trade impact mechanism. Furthermore, the uniqueness of emerging institutional openness platforms such as RCEP (Regional Comprehensive Economic Partnership Agreement) and its impact on trade patterns are often overlooked in the selection of regions for study. More importantly, while it is widely recognized that trade can have a positive impact on human development by improving health (7, 8), promoting income growth (9, 10), and generating education spillovers (11–17), international trade can also exacerbate development gaps between regions due to the uneven distribution of factors, a potentially negative effect that should not be ignored (18).

In view of this, this study focuses on the trade data of medical products between China and RCEP member countries during the period of 2004 to 2023, and employs the stochastic frontier gravity model to measure the export efficiency, and combines the benchmark regression model with the mediation effect model to systematically examine the impact of China’s medical product export efficiency on the human development index (HDI) of RCEP countries and its transmission mechanism. This study aims to fill the gaps in the existing literature, provide new theoretical perspectives and empirical evidences for the in-depth understanding of the complex association between trade in medical products and human development, and provide targeted decision-making references for relevant policy makers and medical product exporters, with a view to promoting the synergistic development of the trade in medical products in the RCEP region, which will ultimately serve the overall enhancement of human well-being.

## 2 Literature review

### 2.1 Related research on medical product trade

In recent years, the total volume of global trade in medical devices has continued to climb, but regional development imbalances have increased, resulting in a poorly sequenced pattern dominated by the core countries and dependent on the peripheral countries (19). This structural contradiction is particularly prominent in the Asia-Pacific region. Studies focusing on the region have shown that technological fault lines and regulatory differences have led to import dependence in many countries (20), with technical barriers to trade showing differential impacts - convergence of standards in the category of “consumer health and safety” can increase the value of ICT medical product imports by 13%, while barriers in the category of “labeling and packaging” can inhibit trade flows instead. Instead, they inhibit trade flows (21). This institutional complexity is evidenced in country-specific cases: Turkey’s medical device import dependence will continue to rise to $1.78 billion (22), India’s exports are hampered by a cumbersome registration process and price controls (23), and COVID-19 is causing Zimbabwe’s supply chain of ARVs to break and triggered price spikes (24). In addition, China has limited pricing power in only 5 out of 21 medical device markets (25), the mean value of technical efficiency in pharmaceutical manufacturing is only 0.832 (26), and shortening the technological distance can significantly enhance product diversity in Asian countries (27). Notably, the synergistic effect of cultural affinity and economic freedom under the RCEP framework provides a new paradigm for regional medical trade resilience (28).

### 2.2 Related research on the human development index

The Human Development Index (HDI), as a core tool for measuring a country’s comprehensive development level, has been widely applied in cross-national research in the fields of health, environment, and socioeconomics in recent years. In terms of health, HDI shows a significant correlation with disease burden. The mortality ratios of breast cancer and gastric cancer are significantly negatively correlated with HDI. Countries with high HDI can improve cancer survival rates due to their medical resource advantages (29, 30). This pattern is also evident in infectious diseases - the tuberculosis mortality rate in low HDI regions of Iran is approximately 2.3 times the national average (31), and the global cataract blindness rate in low-income countries is 11.4 times that of very high-income countries (32). The environmental dimension reveals a development paradox: China’s infrastructure development is positively correlated with HDI, but it also increases CO2 emissions (33). For this reason, scholars have innovatively integrated CO2 emission flows to construct a thermodynamic HDI assessment system (34). Breakthroughs have been made in social policy research: the inclusion of the education index from PISA assessments causes the scores of OECD countries to fluctuate by ±0.12, more accurately reflecting the quality of human capital (35), while fiscal decentralization shows an inverted U-shaped relationship with HDI (36). The emerging perspective of trade linkages has confirmed that trade in high-tech medical products can increase the HDI growth rate of low-income countries by 1.8 times (37).

### 2.3 Innovations, shortcomings and future directions

This study achieves breakthroughs in three aspects: at the theoretical level, it is the first time to verify that the trade efficiency of medical products affects HDI through the complete mediation of basic health service coverage, and to construct a “trade-health-development” transmission chain, which breaks through the limitations of the existing studies that only focus on the direct effect (14); and at the methodological level, it integrates the stochastic frontier model and mediation analysis to quantify the dual-track effects of institutional frictions and technical barriers; The application level proposes differentiated strategies based on different markets in ASEAN, Japan, Korea, and Australia and New Zealand to transform short-term epidemic demand into long-term momentum.

While this study strives to be comprehensive, it has several limitations. First, the possible presence of missing data in the study sample limits our in-depth parsing of heterogeneity within ASEAN member countries to some extent (20). Second, the research framework fails to adequately incorporate the burgeoning digital healthcare revolution in recent years and its potential impact on trade patterns and efficiency (38). Furthermore, at the policy analysis level, there is insufficient exploration of specific policy details that may affect the accessibility of medical products and trade flows, such as drug patent conflicts (39).

Based on the above limitations, future research can be expanded in the following directions: first, combining the intelligent supply chain model to further optimize the efficiency of cross-border logistics of medical products and enhance the smoothness of trade (1); second, attempting to integrate the method of constructing the human development index (HDI) under the thermodynamic perspective, with a view to realizing a two-dimensional comprehensive assessment of the level of medical development and its environmental impact (34); and third, to deeply explore the possible efficiency leap mechanism and its impacts brought by AI technology in the production and trade chain of low-and middle-end medical products. These directions are expected to inject new vitality into the study of trade in medical products and human development.

## 3 Current development status of China’s medical product exports to RCEP countries

### 3.1 The scale of export trade

**Figure 1:**
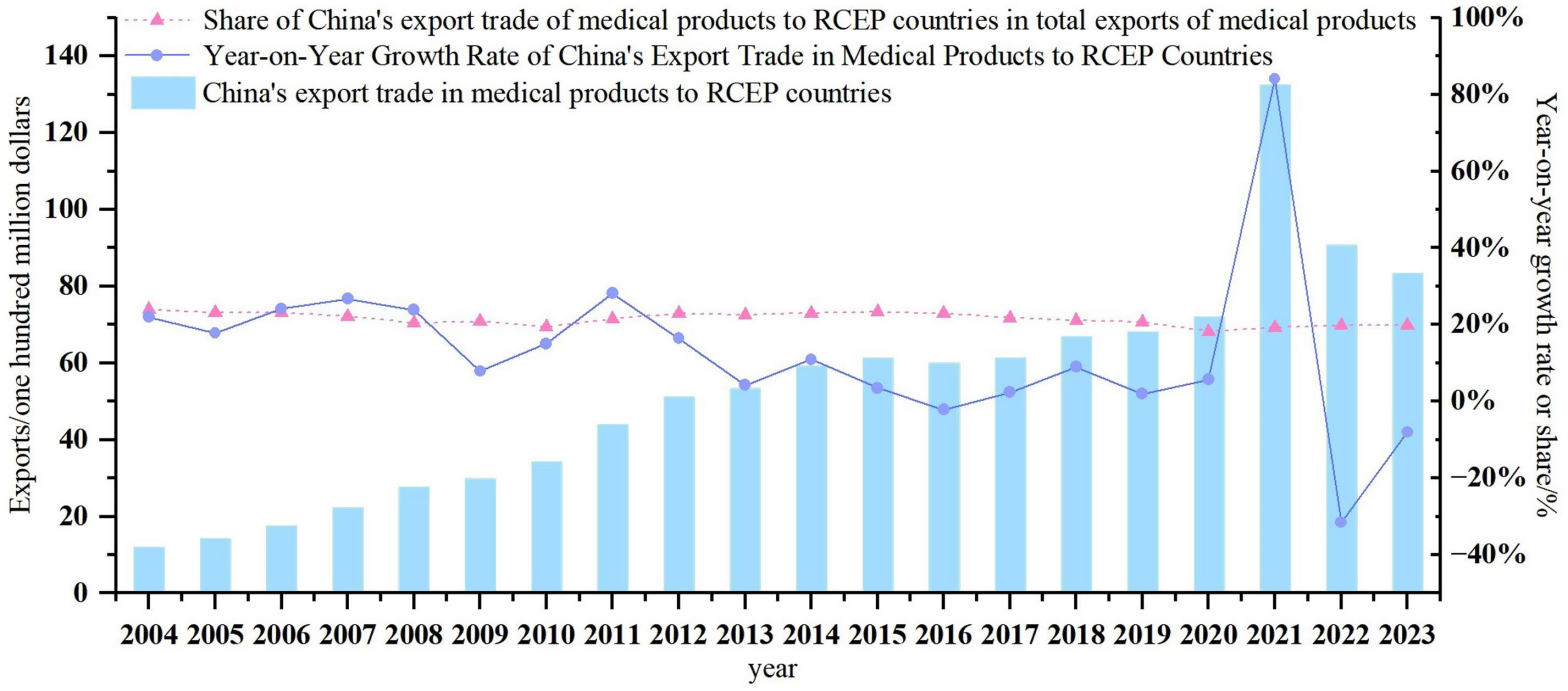
Trends in China’s medical product export trade volume to RCEP countries from 2004 to 2023.

The scale of China’s medical product exports to RCEP countries exhibits phased characteristics. As shown in Figure 1, from 2004 to 2023, export value surged from USD 1.203 billion to USD 8.344 billion, indicating a continuous expansion in overall scale. However, fluctuations in growth rates and proportions reflect dynamic adjustments in regional cooperation and external environmental influences. During the period from 2004 to 2011, the average annual growth rate of export value exceeded 20%, with the proportion consistently maintaining above 20%. This growth was attributed to the deepening of regional cooperation and the rise of China’s medical industry. From 2012 to 2019, influenced by the global economic slowdown and trade protectionism, the average annual growth rate dropped to below 5%, and the proportion declined to around 20%, indicating market saturation and intensified competition. In 2020, the COVID-19 pandemic triggered a surge in demand for medical supplies, leading to an 84% year-on-year increase in China’s export value, reaching USD 13.250 billion. As the pandemic eased and supply chains recovered, export value gradually declined. Overall, the fluctuating growth in China’s medical product exports to RCEP countries, with the proportion stabilizing between 18 and 23%, underscores China’s significant development potential in the RCEP medical product market.

### 3.2 Export industry structure

**Figure 2:**
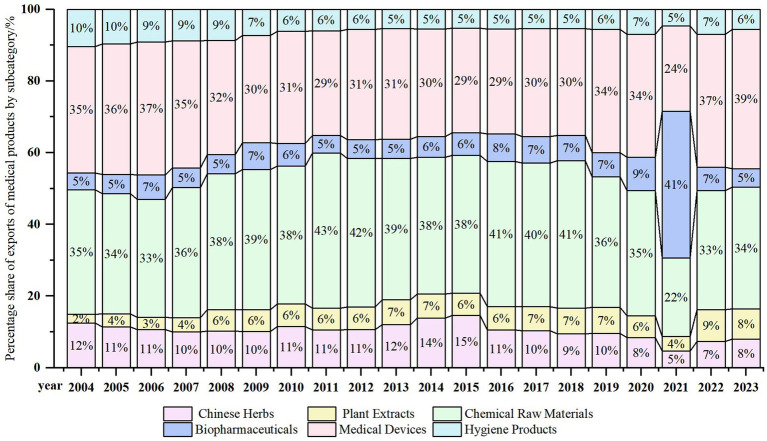
Trends in export value of medical product categories from China to RCEP countries from 2004 to 2023.

China’s medical product exports to RCEP countries are primarily concentrated in areas such as active pharmaceutical ingredients (APIs), medical devices, and biopharmaceuticals, while traditional products like Chinese herbal medicines and plant extracts account for a relatively low proportion. Despite China’s resource advantages in the field of traditional medicine, its international competitiveness still needs to be enhanced. As shown in Figure 2, APIs and medical devices have long held a dominant position, with their combined share consistently maintaining at around 60%. In 2020, influenced by the COVID-19 pandemic, the proportion of biopharmaceuticals rose significantly, reflecting the global surge in demand for vaccines and related biological products. After 2021, as the pandemic gradually eased, the proportion of biopharmaceuticals declined somewhat, while the proportion of medical devices rebounded. Tariff reductions and the harmonization of technical standards under the RCEP framework have provided greater opportunities for China’s exports of high-end medical products. In the future, China should further promote technological innovation in the fields of biopharmaceuticals and medical devices. Meanwhile, through brand building and market expansion, it should enhance the international competitiveness of traditional medicinal products (40).

### 3.3 Export market distribution

**Figure 3:**
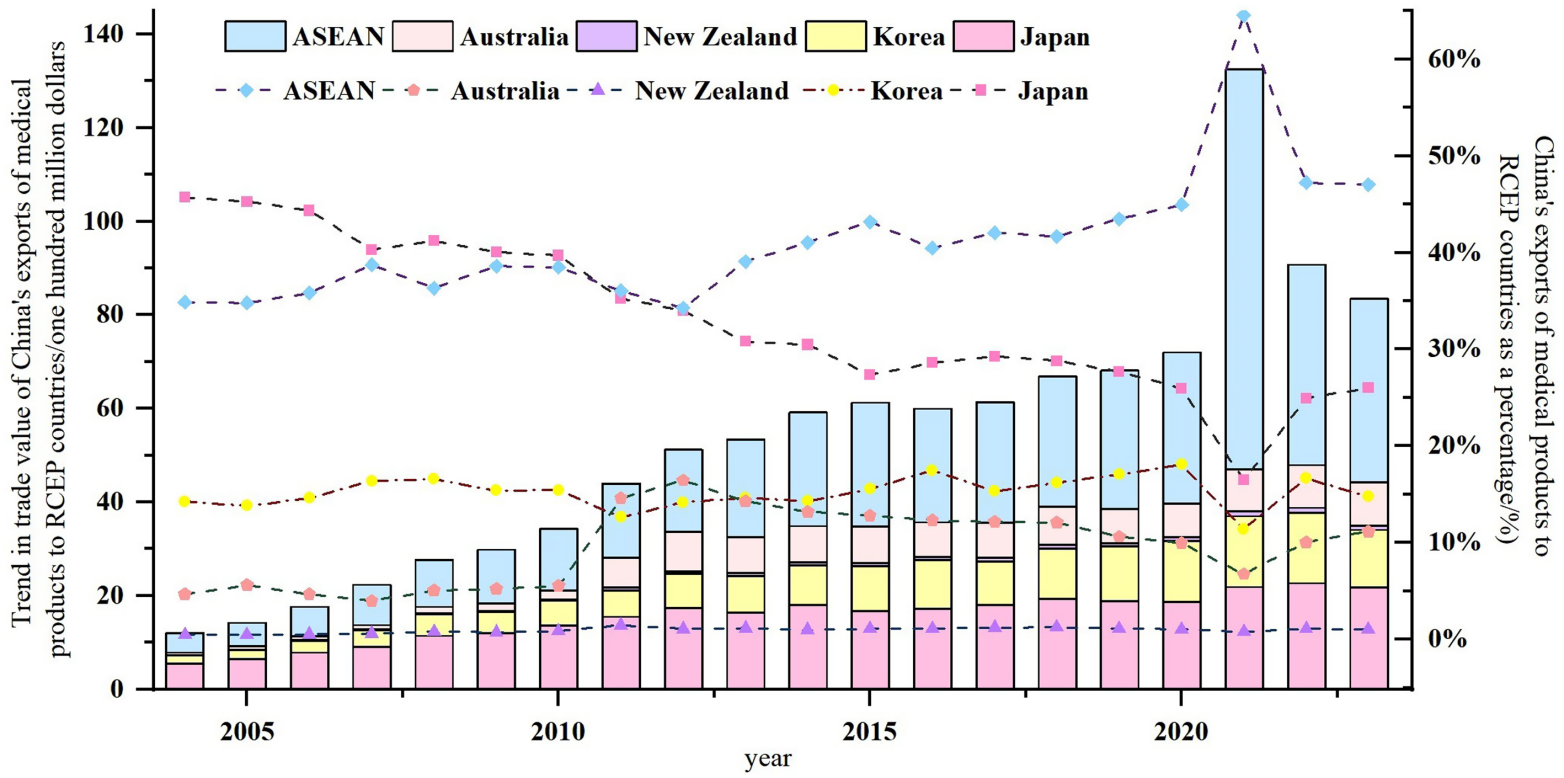
China’s export value and proportion of medical products to RCEP countries from 2004 to 2023.

As shown in Figure 3, the structure of China’s medical product export markets within RCEP countries is predominantly centered around ASEAN, Japan, and South Korea, while Australia and New Zealand hold relatively smaller market shares. In recent years, ASEAN has consistently maintained a trade volume proportion ranging from 30 to 45%, with Japan and South Korea ranking second and third, respectively. In 2021, influenced by the COVID-19 pandemic, the proportion of China’s medical product exports to ASEAN surged to 64.53%. The implementation of the RCEP agreement has further propelled the expansion of China’s market share for medical products in ASEAN. Japan’s share has continued to shrink, potentially due to its strengthening of technical barriers and the “supply chain reshoring” policy; South Korea’s proportion has remained relatively stable with minor fluctuations. China should strengthen trade cooperation with ASEAN, Japan, and South Korea, while simultaneously exploring and expanding markets in Australia and New Zealand to achieve export diversification.

## 4 Model construction and data sources

### 4.1 Model construction

#### 4.1.1 Specification of the stochastic frontier gravity model

The combination of the stochastic gravity model and stochastic frontier analysis provides a powerful tool for exploring the efficiency of medical product exports. The model distinguishes the error term into random noise and efficiency loss, and accurately quantifies the actual and potential gap between China’s exports to RCEP countries. By introducing gravitational factors such as economic size and population, we not only assess the determinants of trade volume, but also analyze in depth the specific causes that lead to trade efficiency losses. This approach allows us to go beyond simple correlation analysis and touch upon the deeper causes of efficiency losses. Therefore, this study combines the characteristics of China’s medical product export trade, selects the factors affecting China’s medical product exports to RCEP countries, and sets up a stochastic frontier gravity model of the following form (Equation 1):


(1) $$\begin{array}{r} \begin{array}{l} \ln\mathrm{EX}P_{\mathrm{ijt}}=\beta_{0}+\beta_{1}\ln\mathrm{PO}P_{\mathrm{it}}+\beta_{2}\ln\mathrm{PO}P_{\mathrm{jt}}+\\ \beta_{3}\ln\mathrm{PGD}P_{\mathrm{it}}+\beta_{4}\ln\mathrm{PGD}P_{\mathrm{jt}} \end{array}\\ \begin{array}{l} +\beta_{5}\ln\mathrm{DIS}T_{\mathrm{ij}}+\beta_{6}\mathrm{BO}R_{\mathrm{ij}}+\\ \beta_{7}\mathrm{LAN}G_{\mathrm{ij}}+\beta_{8}\text{FT}A_{\mathrm{ij}}+\nu_{\mathrm{ijt}}-\mu_{\mathrm{ijt}} \end{array} \end{array}$$

Here, *i* represents China, and *j* represents the RCEP member countries. The dependent variable $\mathrm{EX}P_{\mathrm{ijt}}$ is the export value of medical products from China to RCEP member country *j* in period *t*.$\mathrm{PO}P_{\mathrm{it}}$ and $\mathrm{PO}P_{\mathrm{jt}}$ respectively denote the population sizes of China and RCEP member country *j* in period *t*. $\mathrm{PGD}P_{\mathrm{it}}$ and $\mathrm{PGD}P_{\mathrm{jt}}$ respectively represent the per capita GDPs of China and country *j* in period *t*. $\mathrm{DIS}T_{\mathrm{ij}}$ refers to the straight-line distance between the capitals of the two countries, with data sourced from the World Development Indicators (WDI). $\mathrm{BO}R_{\mathrm{ij}}$ is a dummy variable indicating whether China and RCEP member country *j* share a common border. $\mathrm{LAN}G_{\mathrm{ij}}$ is a dummy variable indicating whether China and RCEP member country *j* share a common language, the data is sourced from the CEPII database. $\text{FT}A_{\mathrm{ij}}$ indicates whether the two countries have signed a Free Trade Agreement, and the data is sourced from the China Free Trade Zone Service Network. $v_{\mathrm{ijt}}$ is the random error term, and $\mu_{\mathrm{ijt}}$ is the inefficiency term.

When constructing the trade inefficiency model, this paper draws on the methodology of Gulinar et al. (41), incorporating healthcare factors and political institutional factors as explanatory variables into the stochastic frontier gravity model (Equation 2).


(2) $$\begin{array}{l} u_{\mathrm{ijt}}=\delta_{0}+\delta_{1}GI_{\mathrm{jt}}+\delta_{2}TB_{\mathrm{jt}}+\delta_{3}GS_{\mathrm{jt}}+\delta_{4}TF_{\mathrm{jt}}+\delta_{5}IF_{\mathrm{jt}}+\\ \delta_{6}FF_{\mathrm{jt}}+\delta_{7}\mathrm{PH}E_{\mathrm{jt}}+\delta_{8}\mathrm{BH}S_{\mathrm{jt}}+\varepsilon_{\mathrm{ijt}} \end{array}$$

In the above equation, $GI_{\mathrm{jt}}$ denotes the government integrity level of RCEP member countries; $TB_{\mathrm{jt}}$ represents the tax burden of country *j* in period *t*, $GS_{\mathrm{jt}}$ represents the government expenditure of country *j* in period *t*, $TF_{\mathrm{jt}}$, $IF_{\mathrm{jt}}$, and $FF_{\mathrm{jt}}$ respectively represent the trade freedom, investment freedom, and financial freedom of country *j*, the economic freedom indices in this paper are all sourced from The Heritage Foundation in the United States (The Heritage Foundation). $\mathrm{PH}E_{\mathrm{jt}}$ and $\mathrm{BH}S_{\mathrm{jt}}$ represent the proportion of private health expenditure and the coverage rate of essential health services in country *j* during period *t*, with the data sourced from the World Development Indicators (WDI).

#### 4.1.2 Specification of the benchmark regression model

A two-way fixed-effects model designed to remove unobservable heterogeneity interference. The model introduces individual and time fixed effects simultaneously on top of the standard fixed effects to precisely control the influence of cross-country individual characteristics and time-varying factors. Compared with other models, it can effectively isolate the net effect of the core explanatory variables on the HDI, improve the estimation accuracy, and provide a reliable basis for analyzing the complex association between the export efficiency of medical products and the HDI. Therefore, the two-way fixed effects model constructed in this paper is as follows (Equation 3).


(3) $$\begin{array}{l} \mathrm{HD}I_{\mathrm{jt}}=\alpha_{0}+\alpha_{1}\mathrm{TE}F_{\mathrm{jt}}+\alpha_{2}TF_{\mathrm{jt}}+\alpha_{3}FF_{\mathrm{jt}}+\\ \alpha_{4}PS_{\mathrm{jt}}+\alpha_{5}GE_{\mathrm{jt}}+\delta_{t}+\mu_{j}+\varepsilon_{\mathrm{jt}} \end{array}$$

Here, $\mathrm{HD}I_{\mathrm{jt}}$ represents the Human Development Index of country *j* in year *t*, with the data sourced from the United Nations Development Program (UNDP). $\mathrm{TE}F_{\mathrm{it}}$ represents the trade efficiency of China’s medical product exports to RCEP countries. Control variables $TF_{\mathrm{jt}}$, $FF_{\mathrm{jt}}$, $PS_{\mathrm{jt}}$, and $GE_{\mathrm{jt}}$, respectively represent a country’s trade freedom, financial freedom, government stability, and government expenditure. $\mu_{j}$ represents the individual fixed effects that do not change over time for country *j*, $\delta_{t}$ controls for the time-fixed effects, and $\varepsilon_{\mathrm{jt}}$ denotes the random disturbance term.

#### 4.1.3 Specification of the mediation effect model

In order to test whether there is a mediating effect between the export efficiency of medical products and the Human Development Index (*HDI*), we introduce a mediating effects model (Equations 4 and 5). The model aims to explore whether health service coverage plays a mediating role in the relationship, i.e., whether increased export efficiency contributes to the *HDI* by improving health service coverage. By sequentially testing the total, direct and indirect effects, we are able to gain a clearer understanding of the specific paths of action between the variables.


(4) $$\begin{array}{l} \mathrm{BH}S_{\mathrm{jt}}=\beta_{0}+\beta_{1}\mathrm{TE}F_{\mathrm{jt}}+\beta_{2}TF_{\mathrm{jt}}+\beta_{3}FF_{\mathrm{jt}}+\\ \beta_{4}PS_{\mathrm{jt}}+\beta_{5}GE_{\mathrm{jt}}+\delta_{t}+\mu_{j}+\varepsilon_{\mathrm{jt}} \end{array}$$

(5) $$\begin{array}{l} \mathrm{HD}I_{\mathrm{jt}}=\gamma_{0}+\gamma_{1}\mathrm{TE}F_{\mathrm{jt}}+\gamma_{2}\mathrm{BH}S_{\mathrm{jt}}+\gamma_{3}TF_{\mathrm{jt}}+\gamma_{4}FF_{\mathrm{jt}}+\\ \gamma_{5}PS_{\mathrm{jt}}+\gamma_{6}GE_{\mathrm{jt}}+\delta_{t}+\mu_{j}+\varepsilon_{\mathrm{jt}} \end{array}$$

We first conduct a regression analysis where the mediating variable *BHS* (Basic Health Services Coverage) serves as the dependent variable, and medical product export trade efficiency acts as the independent variable. Subsequently, we include both medical product export trade efficiency and the mediating variable BHS in the model to examine their joint impact on the Human Development Index (*HDI*). The presence of a mediation effect is determined by evaluating the statistical significance of the coefficients corresponding to $\beta_{1}$, $\gamma_{1}$, and $\gamma_{2}$. The data for BHS is sourced from the World Development Indicators (WDI) database.

### 4.2 Variable description

#### 4.2.1 Dependent variable

The Human Development Index ($\mathrm{HD}I_{\mathrm{jt}}$) is a composite indicator proposed by the United Nations Development Program to measure the level of human development in a country or region. It consists of three dimensions: life expectancy, education level and quality of life. Life expectancy reflects health, education reflects knowledge, and quality of life is about standard of living; the higher the HDI value, the higher the level of human development in the country or region and the happier the people are.

#### 4.2.2 Core explanatory variable

The export trade efficiency ($\mathrm{TE}F_{\mathrm{jt}}$) of medical products from China to RCEP member countries serves as a critical indicator for measuring the gap between China’s actual export performance and its potential capacity in supplying medical products to regional partners. Drawing on the methodology outlined by Gulinar et al. (41), this study utilizes customs-coded data for 19 categories of medical products (as detailed in Table 1) to aggregate the total export value of medical products from China to RCEP member states. The trade inefficiency model is then employed to quantify this efficiency metric.

**Table 1:** Medical products and their corresponding HS codes.

Category	Product	HS code (s)
Pharmaceuticals	Traditional Chinese medicinal materials	1,211, 3,301
	Plant extracts	1,302
	Chemical pharmaceutical raw materials	2,936, 2,937, 2,939, 2,941, 3,003, 3,004
	Biopharmaceuticals	2,938, 3,001, 3,002
Medical devices & hygiene products	Medical devices	9,021, 9,402, 9,018, 9,023, 9,022
	Hygiene & medical supplies	3,005, 3,006

Data source: UN Comtrade Database.

#### 4.2.3 Mediating variable

The Basic Health Services Coverage ($\mathrm{BH}S_{\mathrm{jt}}$) serves as an indicator measuring a country or region’s capacity to deliver essential health services. It reflects the extent and quality of basic medical and health services provided by the government to its citizens. As a mediating variable, BHS quantifies how the export efficiency of medical products from China to RCEP member countries influences the Human Development Index of these nations.

#### 4.2.4 Control variables

Trade Freedom ($TF_{\mathrm{jt}}$) reflects the degree of openness of a country’s trade policy; the higher the value, the lower the trade barriers. Financial Freedom ($FF_{\mathrm{jt}}$) is used to measure the degree of openness of the financial system, the higher the FF value, the more flexible the financial environment. Political stability ($PS_{\mathrm{jt}}$) refers to the stability and orderliness of a country’s political system. Countries that are politically stable are more likely to attract foreign investment and trade, thus promoting economic growth and social development. Government expenditure ($GE_{\mathrm{jt}}$) refers to the total amount of financial outlays by the government to provide public services and implement policies. The level and distribution of government spending directly affects a country’s economic development and social welfare.

### 4.3 Sample selection

This study selects 10 RCEP member countries—Japan, South Korea, Australia, New Zealand, Indonesia, Malaysia, the Philippines, Thailand, Singapore, and Vietnam—as the core sample for the period 2004–2023. Four RCEP members (Brunei, Myanmar, Cambodia, and Laos) were excluded due to severe data gaps. To supplement the sample size, the study also incorporates 15 non-RCEP countries that rank among China’s top trading partners in terms of merchandize export volume: the United States, Germany, India, Russia, Mexico, the United Kingdom, Brazil, Canada, Italy, Saudi Arabia, France, Spain, Turkey, Poland, and Belgium. Missing data points for individual observations were imputed using linear interpolation to ensure data continuity.

## 5 Empirical analysis

### 5.1 Analysis of factors influencing trade efficiency

To examine the impact of human-related factors on trade efficiency, a one-step estimation approach was employed to jointly estimate the stochastic frontier gravity model (SFGM) and the trade inefficiency model. Model γ was statistically significant and approached 1, suggesting a well-specified model (as detailed in Table 2).

**Table 2:** Estimation results of the trade inefficiency model.

Stochastic frontier gravity model (SFGM)			Trade inefficiency model (TIM)		
Variable	Coefficient	*T*-value	Variable	Coefficient	*T*-value
Constant	30.266	1.630	Constant	1.235^**^	2.497
$\mathrm{PO}P_{\mathrm{it}}$	−1.712^*^	−1.891	$GI_{\mathrm{jt}}$	−0.008^***^	−4.467
$\mathrm{PO}P_{\mathrm{jt}}$	0.855^***^	32.922	$TB_{\mathrm{jt}}$	0.026^***^	10.138
$\mathrm{PGD}P_{\mathrm{it}}$	0.957^***^	17.712	$GS_{\mathrm{jt}}$	−0.008^***^	−4.627
$\mathrm{PGD}P_{\mathrm{jt}}$	0.559^***^	12.209	$TF_{\mathrm{jt}}$	0.008^***^	2.783
$\mathrm{DIS}T_{\mathrm{ij}}$	−0.273^***^	−7.362	$IF_{\mathrm{jt}}$	−0.011^***^	−5.543
$\mathrm{BO}R_{\mathrm{ij}}$	0.349^***^	4.111	$FF_{\mathrm{jt}}$	0.011^***^	4.740
$\mathrm{LAN}G_{\mathrm{ij}}$	0.762^***^	7.873	$\mathrm{PH}E_{\mathrm{jt}}$	−0.010^***^	−4.878
$\text{FT}A_{\mathrm{ij}}$	0.237^***^	3.396	$\mathrm{BS}C_{\mathrm{jt}}$	−0.011^***^	−3.322
			$\sigma^{2}$	0.129^***^	12.929
			γ	0.999^***^	11.184
LOG	−191.97				
LR test	278.87				

*, **, and *** denote statistical significance at the 10, 5, and 1% levels, respectively.

The study indicates that trade efficiency is influenced by a multitude of factors, which can be systematically categorized into two dimensions: facilitating and inhibiting factors. In terms of facilitating trade efficiency: Improvements in government integrity and increased government expenditure in trading partner countries create more favorable conditions for trade activities. Higher levels of investment openness in host countries help reduce trade barriers for China’s medical product exports, thereby promoting bilateral trade development. Additionally, investments in the healthcare sector of trading partner countries also exert a positive influence. Specifically, an increased share of private health expenditure and improved Basic Health Services Coverage (BHS) create a more favorable market environment for China’s medical product exports. However, the study also identifies factors that inhibit trade efficiency. Specifically, the tax burden passes the 1% significance test with a positive coefficient, implying that higher tax burdens increase firms’ operational costs, thereby impeding trade efficiency. Notably, while an overall increase in economic freedom generally benefits trade development, excessive liberalization of financial freedom (FF) and trade freedom (TF) may exacerbate risks such as heightened exchange rate volatility and disorderly market competition. These factors can, in the short term, elevate trade friction costs.

### 5.2 Analysis of trade efficiency measurement results

**Figure 4:**
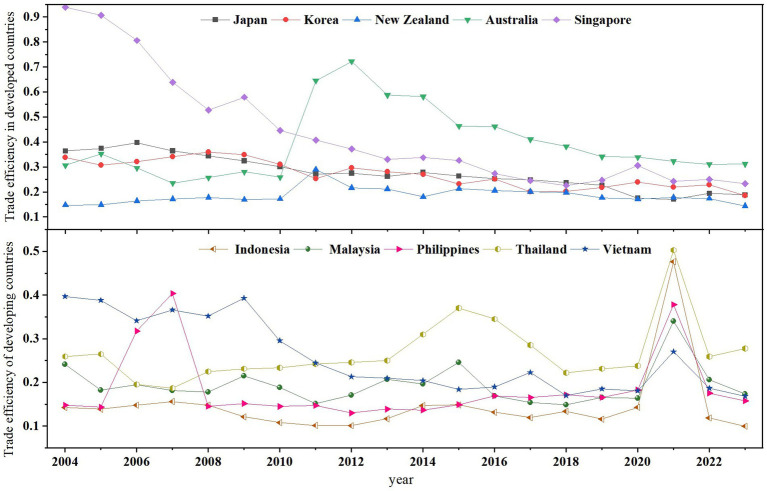
Export trade efficiency of China’s medical products to RCEP countries from 2004 to 2023.

The trade efficiency of China’s medical product exports to RCEP countries exhibits a pronounced characteristic of “divergent evolution.” As shown in Figure 4, the trend of efficiency change shows that the overall efficiency of developed countries is higher, but with a gradual downward trend. Japan’s trade efficiency dropped from 0.36 in 2004 to 0.19 in 2023, and South Korea’s fell from 0.34 to 0.18 over the same period, reflecting market saturation or intensified local competition in these countries. Australia, despite experiencing relatively large fluctuations, has consistently maintained a top-tier position. In contrast, emerging markets generally demonstrate low efficiency with significant volatility. Indonesia’s efficiency has been on a continuous decline since 2004. The Philippines saw its efficiency soar to 0.40 in 2007 due to policy dividends but then dropped back to 0.16. Vietnam’s efficiency has long remained below 0.4, though it experienced a surge in 2021 driven by a sharp increase in demand amid the pandemic. For mature markets like Japan and Australia, the focus should be on high-value-added products and the alignment of technologies to slow down the decline in efficiency. For potential markets such as Indonesia and Vietnam, it is necessary to reduce entry costs through regional supply-chain integration and policy coordination, thereby transforming short-term demand into long-term growth momentum. For volatile markets like the Philippines and Malaysia, a flexible response mechanism needs to be established to balance risks and returns.

### 5.3 Empirical analysis of the benchmark regression model

Considering that there is a lag in the impact of trade efficiency of medical products on national development, this paper adopts the first-order lagged trade efficiency. As shown in Table 3, the trade efficiency of medical products, trade liberalization, financial freedom, the proportion of private health expenditure, and government expenditure all have a significant positive impact on the Human Development Index (HDI) of RCEP countries. The marginal contributions of trade liberalization and financial freedom highlight the crucial roles of market openness and capital mobility. The significance of the proportion of private health expenditure and government expenditure reflects the optimization effect of the synergy between the public and private sectors in the allocation of medical resources. By deepening trade facilitation reforms, strengthening market-opening policies, and balancing public and private medical investments, the development dividends of RCEP in the trade of medical products can be effectively amplified, providing empirical evidence for regional sustainable development.

**Table 3:** Benchmark regression results.

Variable names	Coefficient value	Standard error	*t*-value	*p*-value
Constant term	0.650^***^	0.020	32.18	0.000
$\mathrm{TE}F_{\mathrm{it}}$	0.0141^**^	0.0064	2.21	0.028
$TF_{\mathrm{it}}$	0.0009^***^	0.0002	5.17	0.000
$FF_{\mathrm{it}}$	0.0005^***^	0.0001	5.08	0.000
$PS_{\mathrm{it}}$	0.0165^***^	0.0033	5.03	0.000
$GE_{\mathrm{it}}$	0.0004^**^	0.0002	2.50	0.014
Mean vif	1.84			
*F*	52^***^			
*R* ^2^	0.883			

^*^p < 0.1, ^**^p < 0.05, ^***^p < 0.01.

### 5.4 Empirical analysis of the mediating effect model

Based on the mediating effect test results presented in Table 4, the promoting effect of trade efficiency on the Human Development Index (HDI) of RCEP countries is fully realized through the mediating variable of basic health service coverage.

**Table 4:** Results of the mediating effect test.

Variable names	(1)	(2)
	BHS	HDI
TEF	11.22*** (3.55)	0.00926 (1.42)
BHS	——	0.000438*** (2.77)
Control variables	YES	YES
Individual fixed effects	YES	YES
Time fixed effects	YES	YES
_cons	5.543	0.647^***^
Sample size	190	190
*F*	9^***^	52***
*R* ^2^	0.571	0.889

^*^p < 0.1, ^**^p < 0.05, ^***^p < 0.01.

In Model (1), the coefficient of Trade Efficiency Factor (TEF) on Basic Health Services (BHS) coverage is 11.22 (*p* < 0.01). This indicates that for every one-unit increase in trade efficiency, there is a significant 11.22% improvement in basic health services coverage, thereby verifying the strong positive impact of trade efficiency on the accessibility of health services. In Model (2), while BHS has a statistically significant effect (passing the 1% significance level) on the Human Development Index (HDI), the direct effect of TEF on HDI is not significant. This suggests that the contribution of trade efficiency to enhancing the development index relies entirely on the improvement in health services coverage. The R^2^ values for Model (1) and Model (2) are 0.571 and 0.889, respectively, and the *F*-statistics for both models are highly significant. This demonstrates that the combinations of variables have strong explanatory power for both BHS and HDI, and that the inclusion of individual and time-fixed effects effectively controls for heterogeneity and time trends.

The above empirical findings provide strong support for the existence of an intrinsic link between the export efficiency of medical products, the coverage of basic health services (BHS) and the Human Development Index (HDI), building a coherent theoretical framework. The framework centers on the fact that improved efficiency in the export of essential medical products (e.g., medicines, devices, and consumables) is a key driver for strengthening the health systems of importing countries in RCEP member countries. Efficient trade mechanisms can significantly reduce transaction costs, enhance supply chain reliability, and ensure the timely availability and affordability of key medical inputs within target markets. This improved accessibility of medical products directly contributes to the expansion of basic health services (BHS) coverage, which is the foundation of a functional primary health care system. Increased BHS coverage means that more people have access to preventive, diagnostic, and essential treatment services, which fundamentally improves population health outcomes, a key component of HDI.

In addition, empirical studies confirm the mediating role of BHS coverage in the efficiency of medical product exports in influencing HDI, highlighting the centrality of health system strengthening in the pathway from trade to broader human development. Improved health outcomes (e.g., reduced mortality and morbidity) resulting from better BHS coverage not only directly enhance the health dimension of HDI, but also have positive spillover effects on other dimensions. A healthier population means less lost productivity, which boosts income potential. Improved child health and reduced disease burden also contribute to higher school enrollment and learning outcomes, advancing the education dimension. Thus, the theoretical chain “efficiency in exporting medical products → improved BHS coverage → synergistic gains in HDI (health, education, income)” is validated. The framework positions efficient healthcare trade not only as an economic activity, but also as a powerful tool for promoting health equity and sustainable human development within regional partnerships such as RCEP, aligning trade policy objectives with fundamental public health and development goals.

## 6 Conclusions and recommendations

### 6.1 Conclusion

(1) China’s medical product exports to RCEP countries exhibit an evolutionary pattern characterized by simultaneous scale expansion, structural differentiation, and market concentration. Despite maintaining a long-term growth trend in export trade, its growth rate has shown significant fluctuations due to the combined impacts of external environmental volatility and public health emergencies. From the perspective of industrial structure, chemical raw materials, and medical devices dominate the exports. The export of traditional Chinese medicine products still primarily consists of raw materials, with a scarcity of high-value-added products and inconsistent quality, highlighting the urgency of upgrading the industrial chain (29). The market is concentrated in the region’s core economies, while potential markets are constrained by technological barriers and differences in demand, necessitating the optimization of resource allocation efficiency through regional collaboration.
(2) The dynamic evolution of trade efficiency reflects the dual mechanism of institutional environment and technological capabilities. Economic scale and institutional cooperation serve as the foundation for export expansion, while the high value-added nature of products and supply chain upgrades partially mitigate traditional barriers such as geographical distance. Analysis of trade inefficiency indicates that the governance level and the soundness of the health system in the destination country are crucial for optimizing trade efficiency, whereas excessive liberalization and tax burdens exacerbate friction costs.
(3) The enhancement of China’s medical product export efficiency indirectly promotes the comprehensive development of RCEP countries by strengthening regional health service capabilities. Improved efficiency ensures a stable supply of critical medical resources and, by expanding the coverage of primary healthcare, forms a “trade-health-development” transmission mechanism. This effect highlights the dual economic and public attributes of medical trade, serving as a crucial lever for balancing regional efficiency and equity and achieving sustainable development.

### 6.2 Recommendations

#### 6.2.1 Recommendations for policymakers

Deepen institutional coordination to systematically reduce inefficient barriers (42). Promote mutual recognition of technical standards and sharing of regulatory information within the RCEP framework to establish an efficient and transparent mechanism for cross-border trade (39). For countries with high trade friction, explore the combined model of “technical cooperation + tariff concessions” to balance liberalization and risk prevention and control, so as to unleash long-term trade potential.

Establish a “trade-health” collaborative governance framework to amplify regional public health dividends. Promote the deep integration of medical product trade with health capacity building, and coordinate resource allocation and infrastructure investment through multilateral cooperation mechanisms. Enhance health service coverage through a “material assistance + technical training” model, and establish a closed-loop mechanism of “efficiency-equity-sustainability” to ensure that medical resources are translated into human development outcomes.

#### 6.2.2 Recommendations for exporters

Implement a tiered market strategy to establish a diversified and complementary export structure. For mature markets (such as Japan and South Korea), prioritize the export of high-end medical devices and customized biological preparations, while strengthening technological adaptability and after-sales service systems. For the ASEAN market, reduce entry costs through regional industrial chain integration and expand the supply of inclusive products. For potential markets like Australia and New Zealand, promote the standardization and branding of traditional Chinese medicine.

Strengthening technological innovation and digitalization to enhance the industry chain’s ability to resist risks. Exporters should increase R&D investment in cutting-edge fields such as biopharmaceuticals and precision medical devices, and set up innovation centers in the region through the strategy of “technology going out” to promote knowledge sharing and technology exchange (43). At the same time, the construction of a digital cross-border supply chain platform with real-time logistics tracking and full quality traceability will not only optimize daily operational efficiency, but also significantly improve the emergency response capability to deal with public health incidents (38).

## Data Availability

The original contributions presented in the study are included in the article/supplementary material, further inquiries can be directed to the corresponding author.
